# Increased Photosynthetic Capacity and Energy Status Contribute to Higher Grain Yield in Early Rice

**DOI:** 10.3390/ijms26041508

**Published:** 2025-02-11

**Authors:** Haoran Su, Wenting Wang, Tingting Lu, Wenfei Hu, Junjiang Lin, Weimeng Fu, Yan Liang, Yvxiang Zeng, Guanfu Fu, Jie Xiong, Tingting Chen

**Affiliations:** 1 College of Life Sciences and Medicine, Zhejiang Sci-Tech University, Hangzhou 310018, China; suhaoran0412@163.com (H.S.); 15593823808@163.com (W.H.); 18022557884@163.com (J.L.); 2 State Key Laboratory of Rice Biology and Breeding, China National Rice Research Institute, Hangzhou 310006, China; wangwenting@caas.cn (W.W.); lutingting2024@163.com (T.L.); fuwmeng@163.com (W.F.); liangyan@caas.cn (Y.L.); zengyvxiang@caas.cn (Y.Z.); fuguanfu@caas.cn (G.F.); 3 College of Agronomy, Jilin Agricultural University, Changchun 130118, China

**Keywords:** grain yield, photosynthetic capacity, carbohydrate accumulation, energy status, antioxidant enzymes

## Abstract

As the economy develops and urbanization progresses, the amount of arable land continues to decline. In this context, the cultivation of double-season rice is particularly important for enhancing yield per unit area. However, research on the physiological mechanisms that contribute to high yields in double-season early rice varieties with short growing seasons is still limited. To address this gap, we conducted a field study using two early rice varieties, Zhongzu18 and Yongxian15, to examine their production characteristics, photosynthesis, fluorescence, and energy metabolism. The results indicate that Zhongzu18 has a significantly higher seed-setting rate, grain weight, and total grain yield compared to Yongxian15. Additionally, Zhongzu18 exhibits a higher head rice rate and a lower degree of chalkiness, along with a reduced chalky grain rate. Furthermore, the total dry matter weight and the ratio of panicle weight to total weight for Zhongzu18 were significantly greater than those for Yongxian15. After anthesis, Zhongzu18 also demonstrated a higher leaf net photosynthetic rate and actual fluorescence quantum efficiency compared to Yongxian15. Moreover, the levels of ATP and ATPase, as well as the activities of antioxidant enzymes and the expression of sucrose transport-related genes, were significantly increased in Zhongzu18 plants relative to Yongxian15. We conclude that the enhanced photosynthetic efficiency and energy production in Zhongzu18 lead to more effective assimilation and carbohydrate transport to the grains, resulting in higher grain yields and improved rice quality.

## 1. Introduction

As a crucial global food crop, rice (*Oryza sativa* L.) is vital for maintaining food security worldwide [1]. China, as the largest producer and consumer of rice globally, contributes the highest total yield. Nearly 60% of the world’s population depends on rice as their primary dietary staple [2]. Regions such as the middle-lower Yangtze River basin and southern China possess abundant thermal and solar resources, providing ideal conditions for cultivating double-season rice [3]. Double-season rice yields more total production compared to single-season rice [4]. Therefore, increasing the production of double-cropping rice is vital for boosting total grain output and ensuring food security in China [5]. The early rice harvest, as the first season of annual grain production, helps secure the annual food supply, stabilize the food market, and meet basic food demands [6]. Additionally, the planting area and yield of early rice directly impact the planning of late rice cultivation in the following season, determining the overall rice production for the year [7]. Optimized early rice production can significantly enhance farmers’ incomes through increased annual rice yields.

However, early rice production faces challenges from natural factors such as floods, droughts, low temperatures, and insufficient light, which limit yield increases [8,9,10]. Extended periods of cloudy and low-light conditions during the growth of early rice can negatively affect leaf photosynthesis, leading to a reduction in photosynthetic products [11,12]. This can restrain plant growth and decrease tillering, the number of grains per panicle, and grain weight, ultimately lowering yields. Such conditions are exacerbated in humid climates with frequent rainfall [13]. From a variety perspective, the renewal of early rice varieties in some areas is relatively slow. Many older varieties have limited yield potential and poor stress resistance and struggle to adapt to changing planting conditions and production needs [14]. Additionally, the insufficient promotion and use of high-quality, high-yield varieties also hinder early rice yield improvements [15,16].

According to statistical data, the national early rice yield in 2024 was 5925.4 kg/hm^2^ (395.0 kg/mu), a decrease of 61.6 kg/hm^2^ (4.1 kg/mu) compared to the previous year, representing a decline of 1.0% [17]. Since the early rice sowing season, average temperatures in key producing areas of South China have been close to the long-term averages for this period in recent years. There have been no extreme weather events, and the climate has been more humid than in previous years, which generally benefits sowing, seedling growth, and transplanting of early rice. Thus, there is an urgent need for research into the physiological mechanisms behind high-yield early rice to analyze yield-limiting factors, explore yield potential, and develop appropriate cultivation techniques for higher yields.

In living organisms, the synthesis and accumulation of photosynthetic carbohydrates are closely linked to energy metabolism [18,19]. Together, these processes form the foundation for all life activities and are essential for maintaining normal cell metabolism. Carbohydrate and energy metabolism influence rice yield and quality by affecting assimilation transport, pollen grain germination, pollen tube elongation, and grain filling [20,21]. The elongation of pollen tubes in higher plants relies on energy substances such as NAD(P)H and adenosine ATP, which are produced through glycolysis, the citric acid cycle, and mitochondrial respiration in plastids [22]. Insufficient energy can inhibit pollen germination and tube elongation, significantly impacting the seed-setting rate [23]. Starch, the main component of rice endosperm, accumulates during rice grain development [24]. A lack of assimilates or disruptions in transport can lead to poor starch plastid development in the endosperm, resulting in chalkiness and reduced quality [25]. Achieving high yield, quality, and stress resistance in crops requires adequate energy [26]. However, energy generated through photosynthesis and respiration is limited [27]. Therefore, exploring the mechanisms of photosynthesis and energy metabolism in high-yield rice can help balance plant metabolism by regulating energy levels, providing a solid theoretical foundation for cultivating high-yield, high-quality rice varieties. In this study, we compared grain yield and rice quality between two early rice varieties: Zhongzu18 (known for higher grain yield and superior quality) and Yongxian15 (control). We investigated dry matter production, carbohydrate content (including starch and soluble sugars), photosynthetic rates, fluorescence parameters, chlorophyll content index (SPAD values) indicating leaf senescence, antioxidant enzyme activities, sucrose transport-related gene expressions, and ATP and ATPase levels that characterize energy status. Our aim is to reveal the physiological mechanisms underlying high yield formation in early rice and provide insights for increasing grain yield.

## 2. Results

### 2.1. Grain Yield and Rice Quality

**Figure 1:**
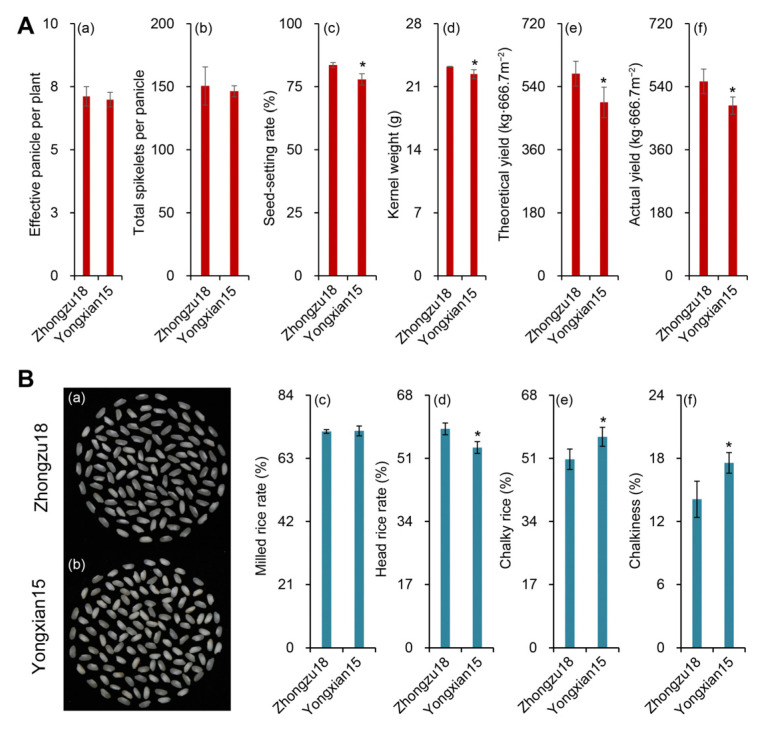
Grain yield and rice quality of two early rice varieties. (**A**) Yield components and actual grain yield: (**a**) panicle numbers; (**b**) grain numbers of each panicle; (**c**) seed-setting rate; (**d**) kernel weight; (**e**) theoretical grain yield; (**f**) actual grain yield. (**B**) Grain quality parameters: (**a**) head rice appearance of Zhongzu18; (**b**) head rice appearance of Yongxian15; (**c**) milled rice rate; (**d**) head rice rate; (**e**) chalky rice; (**f**) chalkiness. Error bars represent ± standard deviation (*n* = 4). Asterisks (*) indicate significant differences at the 0.05 probability level between the two varieties.

As shown in Figure 1A(e,f), both the theoretical and actual yields of Zhongzu18 were significantly higher than those of Yongxian15, with increases of 35.6% and 32.3%, respectively. In terms of yield components, Zhongzu18 had a higher number of effective panicles per plant and grains per panicle compared to Yongxian15 (Figure 1A(a,b)). Additionally, the seed-setting rate and 1000-grain weight for Zhongzu18 were significantly greater (Figure 1A(c,d)). Thus, the yield difference between the two varieties can primarily be attributed to the seed-setting rate and grain weight. The results of the rice quality determination are presented in Figure 1B. The appearance of head rice for both varieties is shown in Figure 1B(a,b). Notably, the head rice rate for Zhongzu18 is significantly higher than that of Yongxian15 (Figure 1B(d)). Furthermore, the chalkiness degree and chalky grain rate of Zhongzu18 are significantly lower than those of Yongxian15 (Figure 1B(e,f)).

### 2.2. Dry Weight Accumulation

**Figure 2:**
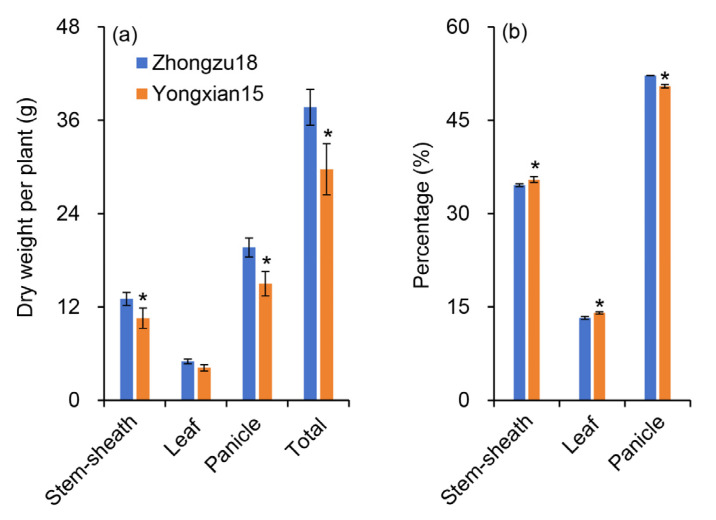
Dry matter accumulation and allocation in rice plants at maturity. (**a**) Dry weights of stems and sheaths, leaves, panicles, and total weight; (**b**) ratios of stem and sheath, leaf, and panicle weights to total dry weight. Error bars represent ± standard deviation (*n* = 4). Asterisks (*) indicate significant differences at the 0.05 probability level between the two varieties.

Figure 2 illustrates the dry weights of plant organs at maturity. The results show that panicle weight was the largest, followed by stem and sheath weight, with leaf weight being the lowest. At maturity, Zhongzu18 had significantly higher dry weights for the panicle, stem, and sheath compared to Yongxian15. Although the dry weight of the leaves of Zhongzu18 was higher, the difference was not statistically significant (Figure 2a). Overall, the total dry matter weight of Zhongzu18 was also significantly greater than that of Yongxian15. The ratio of dry weights for stems and sheaths, leaves, and panicles in Zhongzu18 were significantly lower than those in Yongxian15, while the ratio of panicle dry weight to total dry weight was significantly higher. This suggests that Zhongzu18 more effectively utilizes assimilated substances from its leaves, stems, and sheaths, leading to greater transport to the panicles and conversion into grain yield compared to Yongxian15.

### 2.3. Photosynthesis and Chlorophyll Fluorescence Parameters

**Figure 3:**
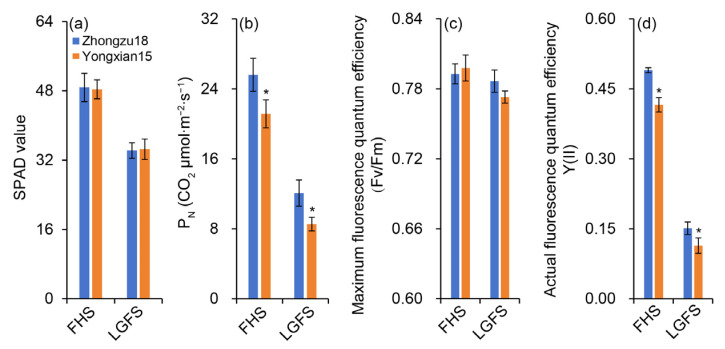
Photosynthesis parameters of rice leaves. (**a**) SPAD value; (**b**) net photosynthetic rate; (**c**) maximum fluorescence quantum efficiency (Fv/Fm); (**d**) actual fluorescence quantum efficiency. FHS, LGFS indicates, respectively, the full heading stage and the late grain-filling stage. Error bars represent ± standard deviation (*n* = 4). Asterisks (*) indicate significant differences at the 0.05 probability level between the two varieties.

The results for photosynthesis-related parameters measured at the full heading stage (FHS) and late grain-filling stage (LGFS) indicate no significant difference in SPAD values between the two varieties (Figure 3a). However, the net photosynthetic rate of the flag leaves was significantly higher in Zhongzu18 than in Yongxian15 during both measurement periods (Figure 3b).

Regarding chlorophyll fluorescence parameters, Zhongzu18 exhibited a slightly lower maximum fluorescence quantum efficiency (Fv/Fm) than Yongxian15 at the FHS, but was slightly higher at late grain filling, with no significant difference (Figure 3c). The actual fluorescence quantum efficiency (YII) was significantly higher in Zhongzu18 than in Yongxian15 during both measurement periods (Figure 3d). This indicates that while the maximum potential for photosynthesis was similar for both varieties, Zhongzu18 demonstrated significantly superior actual performance.

### 2.4. Carbohydrate Content

**Figure 4:**
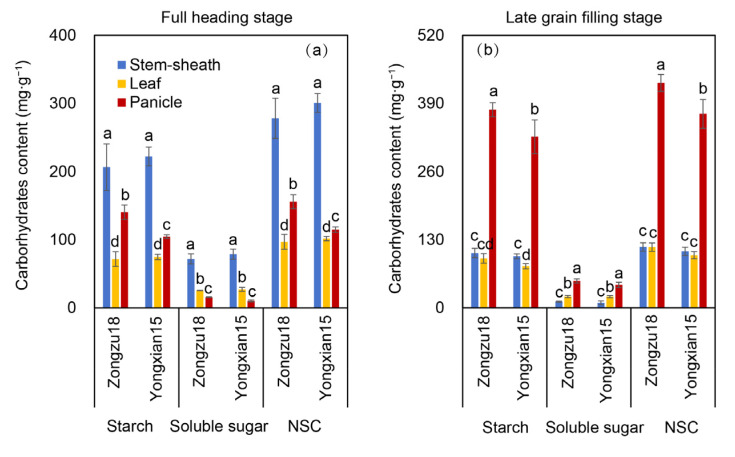
Starch, soluble sugar, and non-structural carbohydrate (NSC) contents in two early rice varieties. (**a**) Carbohydrate content in sheaths and stems, leaves, and panicles at the FHS; (**b**) carbohydrate content in sheaths and stems, leaves, and panicles at the LGFS. Error bars represent ± standard deviation (*n* = 4). Different letters indicate significant differences at the 0.05 probability level between the two varieties.

Overall, rice plants exhibited higher starch levels compared to soluble total sugar content, as illustrated in Figure 4. During the FHS stage, the stems and sheaths of both rice varieties contained significantly elevated soluble sugar concentrations relative to leaves. Among the three organs, panicles displayed the lowest soluble sugar content, with no notable variation between varieties (Figure 4a). Starch accumulation in stems and sheaths markedly exceeded that in panicles, while leaves showed minimal starch content. Except for Zhongzu18’s panicles, which had significantly higher starch levels than Yongxian15, no significant differences were observed in starch content across leaves, stems, and sheaths between the two varieties. The non-structural carbohydrate (NSC) content followed a pattern similar to starch distribution.

At the LGFS stage, panicles of both varieties accumulated significantly more starch than stems and sheaths, with leaves remaining the lowest. Apart from Zhongzu18’s panicles showing notably higher starch content than Yongxian15, starch levels in leaves, stems, and sheaths did not differ significantly between the two cultivars (Figure 4b). Soluble sugar content in panicles was substantially higher than in leaves, while stems and sheaths recorded the lowest values. No significant inter-varietal differences in soluble sugar content were detected (Figure 4b). The NSC content trend aligned closely with starch distribution patterns.

### 2.5. Gene Expression of SUT

**Figure 5:**
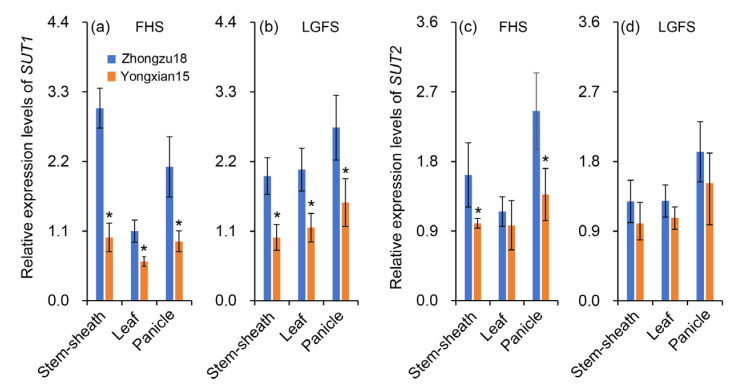
Relative mRNA levels of *sucrose transporter* (*SUT*) genes in two early rice varieties. (**a,b**) Gene expression of *SUT1* in sheaths and stems, leaves, and panicles at the full heading stage (FHS) and late grain-filling stage (LGFS); (**c**,**d**) Gene expression of *SUT2* in sheaths and stems, leaves, and panicles at the FHS and the LGFS. Error bars represent ± standard deviation (*n* = 4). Asterisks (*) indicate significant differences at the 0.05 probability level between the two varieties.

Figure 5 illustrates the gene expression characteristics of *SUT1* and *SUT2* in the leaves, panicles, and stem sheaths of rice at both the FHS and LGFS. The results indicate that the expression level of *SUT1* in Zhongzu18 is significantly higher than in Yongxian15 across all tissues (leaves, panicles, stems, and sheaths) during the same period (Figure 5a,b). For *SUT2*, gene expression in leaves, panicles, stems, and sheaths at the FHS is higher in Zhongzu18 compared to Yongxian15; however, this difference is only statistically significant in panicles, stems, and sheaths (Figure 5c). At the LGFS, *SUT2* gene expression remains higher in Zhongzu18 across all tissues, but the differences are not statistically significant (Figure 5d).

### 2.6. ATP and ATPase Content

**Figure 6:**
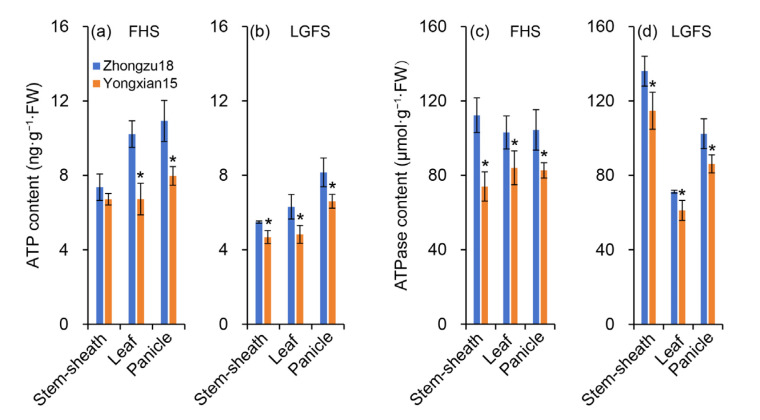
ATP and ATPase contents in two early rice varieties. (**a**,**b**) ATP content in sheaths and stems, leaves, and panicles at the full heading stage (FHS) and the late grain-filling stage (LGFS); (**c**,**d**) ATPase content in sheaths and stems, leaves, and panicles at the FHS and the LGFS. Error bars represent ± standard deviation (*n* = 4). Asterisks (*) indicate significant differences at the 0.05 probability level between the two varieties.

As shown in Figure 6a, the ATP content in the leaves, panicles, stems, and sheaths of rice plants at the FHS is higher in Zhongzu18 than in Yongxian15. The difference in ATP content between the varieties in stems and sheaths is not significant; however, there is a significant difference in ATP content for leaves and panicles. At the LGFS, ATP content in stems sheaths, leaves, and panicles is significantly higher in Zhongzu18 compared to Yongxian15 (Figure 6b).

The ATPase content in leaves, panicles, stems, and sheaths is similar within each rice variety at the FHS, but significant differences exist between the varieties. Specifically, ATPase content is significantly higher in Zhongzu18 than in Yongxian15 (Figure 6c). At the LGFS, ATPase content is higher in stems and sheaths compared to panicles and leaves, and it is significantly greater in Zhongzu18 across all plant parts (Figure 6d).

### 2.7. Antioxidant System Enzyme Activity and MDA Content

**Figure 7:**
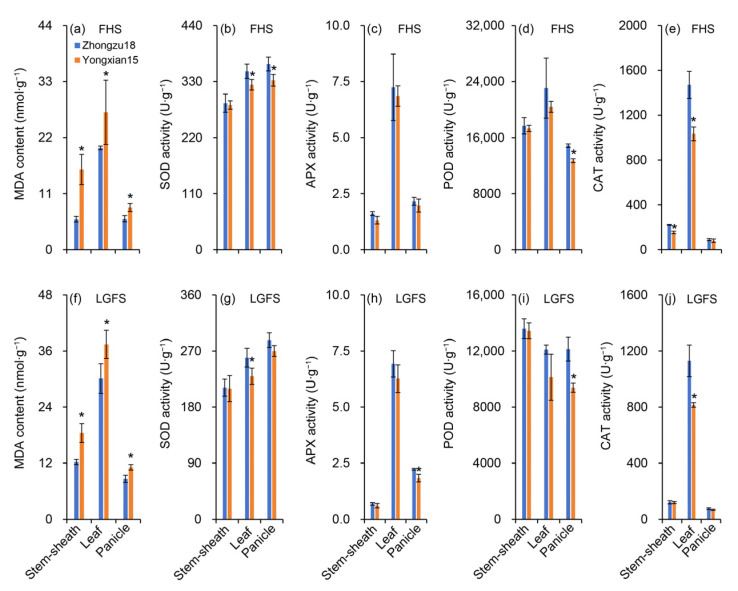
Antioxidant-related enzyme activities and malondialdehyde (MDA) contents in two early rice varieties. (**a**,**f**) MDA content in sheaths and stems, leaves, and panicles at the full heading stage (FHS) and the late grain-filling stage (LGFS); (**b**,**g**) superoxide dismutase (SOD) activity in sheaths and stems, leaves, and panicles at the FHS and the LGFS; (**c**,**h**) ascorbate peroxidase (APX) activity in sheaths and stems, leaves, and panicles at the FHS and the LGFS; (**d**,**i**) peroxidase (POD) activity in sheaths and stems, leaves, and panicles at the FHS and the LGFS; (**e**,**j**) catalase (CAT) activity in sheaths and stems, leaves, and panicles at the FHS and the LGFS. Error bars represent ± standard deviation (*n* = 4). Asterisks (*) indicate significant differences at the 0.05 probability level between the two varieties.

Figure 7a,f show the changes in malondialdehyde (MDA) content in the leaves, grains, stems, and sheaths of rice at both the FHS and LGFS. Overall, MDA content is higher in the LGFS compared to the FHS. Among different plant organs, MDA content is highest in leaves, followed by stems and sheaths, with panicles having the lowest levels. Additionally, MDA content in leaves, grains, stems, and sheaths is significantly lower in Zhongzu18 than in Yongxian15 during both measurement periods. Superoxide dismutase (SOD) enzyme activity is significantly higher in Zhongzu18 compared to Yongxian15 in leaves and panicles at the FHS, while there is no significant difference in stems and sheaths (Figure 7b). In the LGFS, only the SOD enzyme activity in the leaves of Zhongzu18 is significantly greater than in Yongxian15 (Figure 7g). Ascorbate peroxidase (APX) enzyme activity is generally higher in Zhongzu18 than in Yongxian15 at both measurement stages, but significant differences are only observed in panicles during the LGFS (Figure 7c,h). At both measurement periods, peroxidase (POD) enzyme activity in Zhongzu18 is significantly higher than in Yongxian15 only in panicles. Although POD activity is greater in leaves, stems, and sheaths of Zhongzu18, the differences are not statistically significant (Figure 7d,i). Catalase (CAT) enzyme activity is significantly higher in Zhongzu18 than in Yongxian15 in leaves, stems, and sheaths at the FHS, and only in leaves at the LGFS. There are no significant differences in CAT enzyme activity between varieties in other organs during either measurement period (Figure 7e,j).

## 3. Discussion

Adverse climatic conditions are external factors that often lead to low and unstable yields in early rice production [28]. Furthermore, the potential of high-yielding varieties, which possess excellent genetic traits, has not been fully realized due to inadequate cultivation techniques. This underuse of hybrid vigor is a significant factor limiting yield improvements. Many researchers note that modern high-yielding rice varieties typically have a growth period of around 120 days. Shortening this growth period can result in insufficient vegetative development, significantly reducing yield potential. For double-season rice, widely transplanted early and late varieties generally have growth periods ranging from 115 to 130 days. Under optimal high-yield management conditions, a single season of rice can yield between 8 and 9 tons per hectare [29]. In this study, the high-yielding variety Zhongzu18 has a relatively short growth period of about 110 days. In comparison, the control variety Yongxian15 has a similar growth period, but Zhongzu18 achieves a yield of up to 8.7 tons per hectare, representing a theoretical yield that is 35.6% higher than that of the control (Figure 1A). Research indicates that the length of the rice growth period is closely related to yield, with shorter growth varieties generally having lower yield potential compared to medium- and long-growth varieties. Rice plants capture solar radiation for photosynthesis, which is crucial for biomass accumulation and yield formation [30]. A shorter growth period means less available solar radiation each season, potentially leading to reduced biomass accumulation and grain yield. Therefore, the growth period alone is not the primary reason for the yield differences observed between the two varieties in this study. Usually, the number of grains per panicle and grain weight of rice are negatively correlated, because the total amount of nutrients that can be distributed during the growth of rice was relatively fixed and limited, while there is a potential competing relationship between yield components. It is gratifying that yield components of Zhongzu18 are synergistically improved in our study when compared with Yongxian15. Doubtlessly, modern varieties with high yield properties tend to have coordinated yield components owing to genetic selection and improvement, strong physiological support (developed root system, high efficiency of photosynthesis, carbohydrates accumulation, nutrient transport, etc.), and precise cultivation management [24,31].

In terms of dry matter accumulation, high-yielding varieties at maturity show significantly higher dry matter levels in leaves, panicles, stems, and sheaths compared to the control variety (Figure 2a). This greater accumulation of dry matter provides the necessary materials for grain filling and final yield. Additionally, the distribution of dry matter changes at maturity. Zhongzu18 shows a significant decrease in the ratio of stem and sheath and leaf dry weight to total weight, while the percentage of panicle dry weight increases significantly compared to Yongxian15 (Figure 2b). This suggests that more photosynthetic assimilates from the leaves, stems, and sheaths of Zhongzu18 are transported to the grains, effectively converting into yield. Actually, it can also be inferred from the change tendency of carbohydrates content (Figure 4). Among carbohydrates, sucrose is the primary form transported over long distances from source to sink tissues in rice. Plants fix atmospheric carbon dioxide through photosynthesis to produce triose phosphate, which is then synthesized into sucrose or starch. A significant amount of this synthesized sucrose is loaded into the phloem for transport to various sink tissues, supporting plant growth and development [32]. Sucrose transporters (*SUTs*) are crucial for the loading, transport, and unloading of sucrose [33]. In fact, there are five *SUT* genes that act collaboratively to regulate the distribution of assimilates in rice plants. Nevertheless, *SUT1* and *SUT2* have been extensively documented as the most important ones in sucrose transportation [34]. Our study measured these two gene expression levels, confirming that the sucrose transport-related gene *SUT1* plays an important regulatory role in assimilate loading and unloading during the grain-filling period (Figure 5a,b). *SUT2* appears to be more active in the early stage of grain filling but shows decreased activity in the later stages (Figure 5c,d). This aligns with previous findings that *OsSUT2* is mainly expressed in panicles before flowering [35]. Consequently, the higher expression of sucrose transport-related genes in the stems, sheaths, and leaves of Zhongzu18 facilitates the loading and long-distance transport of sucrose, while increased expression in the panicles aids in sucrose unloading, leading to greater carbohydrate accumulation in the grains. This explains why Zhongzu18 exhibits a significantly higher seed-setting rate and grain weight compared to the control variety (Figure 1A(c,d)).

The formation of high yields in rice is closely linked to solar radiation interception, photosynthetic efficiency, and energy distribution. Research has shown that dry matter production after heading contributes about 70% to grain yield, making biomass accumulation during this stage critical for achieving high yields [36]. Studies have also indicated that leaf greenness after heading can enhance dry matter production during grain filling, improving grain filling and yield [37]. In our study, while there was no significant difference in leaf color or SPAD value between the two varieties after heading, the net photosynthetic rate of the high-yielding variety Zhongzu18 significantly increased (Figure 3a,b). This suggests that both varieties have similar light absorption during photosynthesis, but Zhongzu18 has a higher light energy utilization rate. Despite no significant difference in the Fv/Fm of PSII between the two varieties, Zhongzu18 shows a significant advantage in actual photochemical quantum yield (Figure 3c,d). Additionally, the ATP and ATPase contents in the leaves, panicles, stems, and sheaths of Zhongzu18 were significantly higher than those of Yongxian15 (Figure 6). This indicates that the overall energy level of the high-yielding variety is greater, which supports starch synthesis and other physiological processes, ultimately benefiting yield formation (Figure 4).

**Figure 8:**
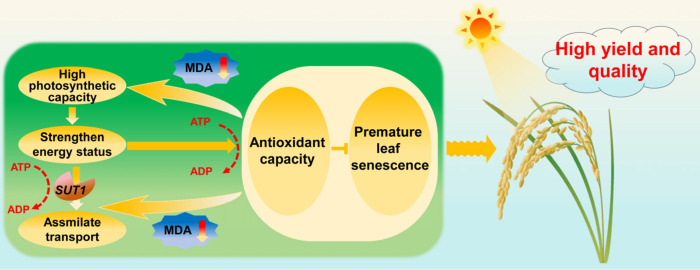
Putative pathway for high yield and quality formation in the early rice variety Zhongzu18. The higher antioxidant enzyme activity in the high-yielding variety Zhongzu18 helps clear peroxides from various plant organs, reducing malondialdehyde (MDA) accumulation. This enhancement improves photosynthetic capacity and strengthens energy status, while also promoting assimilate transport. Additionally, the high photosynthetic capacity and improved energy status in Zhongzu18 provide a positive feedback effect to enhance the plant’s antioxidant capacity, delaying leaf senescence. Increased expression of sucrose transporter (*SUT*) genes, driven by strengthened energy status, further enhances assimilate transport. Consequently, the accumulation of photosynthetic assimilates after heading and the slow leaf senescence during the grain-filling period both contribute to increased yield in Zhongzu18. In the figure, yellow pointed arrow indicates facilitation, yellow blunt-ended arrow indicates inhibition, and red arrow indicates decline.

We also investigated the reasons behind efficient light energy utilization in Zhongzu18. Generally, photosynthetic capacity declines during grain filling, while respiration continues to support vital functions, leading to the production of reactive oxygen species (ROS) such as superoxide anions and hydrogen peroxide in plant cells. Increased ROS can intensify lipid peroxidation, resulting in MDA production as a byproduct [38,39]. In our study, MDA levels in the high-yielding variety were significantly lower than in the control, which correlates with its higher antioxidant enzyme activity, particularly SOD activity. Accumulation of oxidative substances can adversely affect photosynthesis and assimilate transport, reducing carbohydrate synthesis and yield. Additionally, oxidative substances may interact with nutrients in rice grains, negatively impacting quality by reducing protein and starch content. As shown in Figure 8, the higher antioxidant enzyme activity in Zhongzu18 helps clear peroxides in various plant organs, reducing MDA accumulation and improving photosynthetic efficiency and assimilate transport. The high photosynthetic efficiency and energy metabolism levels of Zhongzu18 also provide a positive feedback effect to enhance the plant’s antioxidant properties, delaying leaf senescence and maintaining vitality. Therefore, the combination of high net photosynthetic rates after heading and slow leaf senescence during the grain-filling period contributes to increased yields in early rice varieties with shorter growth periods. To boost the yield of double-season early rice, it is essential to enhance photosynthetic efficiency and energy status during the grain-filling period through cultivation practices or genetic improvements [40]. Modern biotechnology and breeding methods should be applied to cultivate rice varieties with high photosynthetic efficiency genes. These varieties are expected to possess characteristics such as large leaf area, high chlorophyll content, and strong photosynthetic enzyme activity, which would enable them to absorb and utilize light energy more effectively. Also, cultivation measures, including adequate fertilizer and water supply, etc., should be adopted synchronized.

## 4. Materials and Methods

### 4.1. Materials and Experimental Design

The study was carried out at the experimental base of the China National Rice Research Institute in Fuyang City (30.30′ N, 120.2′ E, 11 m above sea level) during the 2022–2023 growing season. Two early indica rice cultivars, Zhongzu 18 and Yongxian 15 (conventional early rice), commonly cultivated in the middle and lower Yangtze River basin, were selected for evaluation. Seeds underwent a 48 h soaking process in distilled water at 35 °C, followed by a 24 h germination period prior to sowing on 4 April. Seeds were uniformly distributed on paddy soil beds and covered with plastic film to shield seedlings from low-temperature stress. Films were later removed to prevent overheating as temperatures increased. Thirty days post-sowing, seedlings were manually transplanted at a density of two seedlings per hill, with 20 cm spacing between rows and hills.

The experimental design consisted of three replicates per variety, each occupying a 25 m^2^ plot. The soil type was classified as purple-blue mud, containing 36.9 g/kg of organic matter, 2.73 g/kg of total nitrogen, 0.60 g/kg of total phosphorus, and 20.1 g/kg of total potassium. The fertilization amounts were 225 kg/hm^2^ of nitrogen, 112 kg/hm^2^ of phosphorus pentoxide, and 225 kg/hm^2^ of potassium chloride. Nitrogen application was split into base (50%), tillering (30%), and panicle (20%) stages, whereas potassium was evenly divided between base and panicle stages. Phosphorus was applied entirely as a basal dose. Field management practices adhered to standardized high-yield rice cultivation protocols.

### 4.2. SPAD Value, Photosynthetic Rate, and Fluorescence Parameters

Leaf chlorophyll content was quantified using a SPAD-502 Plus chlorophyll meter (SPAD-502 Plus, Konica Minolta Sensing, Inc., Tokyo, Japan). Flag leaves from both varieties were sampled during the FHS and LGFS growth stages, with ten leaves per plot selected from the main stems for analysis.

Gas exchange parameters were measured using a Li-6800 portable photosynthesis system (Li-6800, Li-COR Biosciences Inc., Lincoln, NE, USA). A 2 cm^2^ leaf chamber with 1500 μM/(m^2^·s) light intensity, 400 μM/mol CO_2_ concentration, and 600 μM/s flow rate was employed. Chamber conditions were maintained at 30 °C and 60% relative humidity. Measurements were conducted on three flag leaves per plot, targeting the mid-section (4–6 cm from the leaf tip).

Chlorophyll fluorescence parameters (Fv/Fm and Y(II)) were assessed using a PAM-2500 fluorometer (PAM-2500; Heinz Walz, Effeltrich, Germany). After 30 min of dark adaptation, fluorescence values were recorded from three main-stem flag leaves per plot [41].

### 4.3. Dry Matter Weight

At both FHS and maturity, twelve rice hills per plot were harvested and partitioned into stems, sheaths, leaves, and panicles. The samples were deactivated at 105 °C for 1 h, dried at 80 °C for 48 h until a constant weight was reached, and weighed. Dried tissues were pulverized using a grinder and sieved through a 1 mm mesh for subsequent carbohydrate analysis.

### 4.4. Starch, Soluble Sugar, and Non-Structural Carbohydrate Contents

Total soluble sugars and starch were analyzed via anthrone–sulfuric acid colorimetry [41,42]. Ground samples (0.2 g) were mixed with 10 mL deionized water, heated at 100 °C for 30 min, and centrifuged. Supernatants were pooled after three extraction cycles for soluble sugar quantification. Residual precipitates were oven-dried for starch determination. For starch analysis, dried samples were treated with anthrone–sulfuric acid (0.2% in 98% H_2_SO_4_) and boiled for 15 min, and absorbance was measured at 620 nm. Soluble sugar extracts were gelatinized in boiling water treated with perchloric acid (9.2 M and 4.6 M), and absorbance was measured at 620 nm after anthrone addition. NSC content was calculated as the sum of soluble sugars and starch.

### 4.5. Determination of Antioxidant Enzyme Activity and Malondialdehyde Content

Fresh leaf tissue (0.2 g) was homogenized in liquid nitrogen and extracted with 5 mL phosphate buffer (50 mM, pH 7.0). After centrifugation (10,000× *g*, 15 min, 4 °C), supernatants were assayed for SOD, POD, CAT, and APX activities using established protocols [43,44,45,46]. MDA content was determined via thiobarbituric acid reaction. Homogenates were mixed with trichloroacetic acid, and absorbance at 532 nm, 600 nm, and 450 nm was measured [47]. MDA concentration was calculated as C (μM) = 6.45(A_532_ − A_600_) − 0.56A_450_.

### 4.6. Determination of ATP and ATPase Enzyme Content

Frozen leaves (0.1 g) were homogenized in PBS (0.1 M, pH 7.4) and centrifuged (3000× *g*, 20 min). Supernatants were analyzed for ATP and ATPase levels using ELISA kits (Shanghai Enzyme-linked Biotechnology Co., Shanghai, China) with absorbance read at 450 nm (Multiskan FC, Thermo Fisher Scientific, Shanghai, China).

### 4.7. Gene Expression Determination

Total RNA was isolated from leaves (0.3 g) using TRIpure reagent (Aidlab Biotechnologies, Beijing, China) and reverse-transcribed into cDNA. Quantitative PCR was performed with SYBR Green I (TOYOBO, Shanghai, China) on a real-time thermal cycler (TaKaRa Biotechnology, Dalian, China). Gene-specific primers (Supplementary Table S1) were designed using PRIMER5 [48]. Relative expression levels were calculated via the 2^−ΔΔCT^ method [49].

### 4.8. Yield Components and Rice Quality

At maturity, plot yields were calculated based on harvested area and grain weight. Twelve hills per plot were analyzed for effective panicles per plant, grains per panicle, seed-setting rate, and 1000-grain weight to estimate theoretical yield. Grain quality parameters were assessed by the Ministry of Agriculture’s Rice Quality Testing Center.

### 4.9. Data Analysis

The data were processed using Excel and SPSS 11.5 (IBM Corp., Armonk, NY, USA) for statistical evaluation.

## 5. Conclusions

The results of this study show that the yield of Zhongzu18 is significantly higher than that of the control variety, Yongxian15. This is primarily due to a higher seed-setting rate and 1000-grain weight. Zhongzu18 also demonstrates superior rice quality, characterized by a higher head rice rate and lower levels of chalkiness and chalky grains compared to Yongxian15. At maturity, Zhongzu18 has a greater total dry matter weight than Yongxian15. Additionally, the ratio of dry matter weight in the stems and sheaths, as well as the leaves, is lower in Zhongzu18, while the ratio of panicle weight is higher. At both the heading and late grain-filling stages, Zhongzu18 exhibits significantly higher net photosynthetic rates, actual fluorescence quantum efficiency, and levels of ATP and ATPase compared to the control. Moreover, the antioxidant system in Zhongzu18 is significantly more active than in Yongxian15. This enhanced antioxidant activity correlates positively with its higher photosynthetic efficiency and energy levels, which promote the upregulation of *SUT* gene expression and improve the transport of assimilates, ultimately delaying leaf senescence. Carbohydrate and energy metabolism are key pathways influencing the yield and quality of both varieties. The photosynthetic efficiency, energy production, and accumulation of assimilates in Zhongzu18 facilitate greater transport of effective carbohydrates to the grains, resulting in higher grain yields and improved rice quality.

## Data Availability

The data that support the results of this study are available from the corresponding author, upon reasonable request.

## Supplementary Materials

The following supporting information can be downloaded at: https://www.mdpi.com/article/10.3390/ijms26041508/s1.

## Abbreviations

APX: Ascorbate peroxidase; CAT: Catalase; FHS: Full heading stage; LGFS: Late grain filling stage; MDA: Malondialdehyde; NSC: Non-structural carbohydrate; POD: Peroxidase; ROS: Reactive oxygen species; SOD: Superoxide dismutase.
