# Supplementary material for: Increased Photosynthetic Capacity and Energy Status Contribute to Higher Grain Yield in Early Rice
Source: Int J Mol Sci. 2025 Feb 11;26(4):1508. doi: 10.3390/ijms26041508 (PMC11855855; doi:10.3390/ijms26041508)
Supplement: Supplementary file 1 [file ijms-26-01508-s001.zip › ijms-3418119-supplementary.pdf]

Table S1 Primer sequences used in qRT-PCR.

Gene	Forward (5'-3')	Reverse (5'-3')
SUT1	GGTGGCATGCTGCTATTGTA	GTTAGCTGTGCCAGGTCCAT
SUT2	CCGTTCACCGTTACTCCATC	GAGGCTCTTGCACTGATGCT
